# Characterization of NLRP3 Inflammasome Activation in the Onset of Diabetic Retinopathy

**DOI:** 10.3390/ijms232214471

**Published:** 2022-11-21

**Authors:** Charisse Y-J. Kuo, Jack J. Maran, Emma G. Jamieson, Ilva D. Rupenthal, Rinki Murphy, Odunayo O. Mugisho

**Affiliations:** 1 Buchanan Ocular Therapeutics Unit, Department of Ophthalmology, New Zealand National Eye Centre, Faculty of Medical and Health Sciences, The University of Auckland, Auckland 1023, New Zealand; 2 Department of Medicine, Faculty of Medical and Health Sciences, The University of Auckland, Auckland 1023, New Zealand

**Keywords:** NLRP3, inflammasome, activation, diabetic retinopathy, onset, human, retina, vitreous

## Abstract

The aim of this study was to characterize the role of nucleotide-binding oligomerization domain- (NOD-) like receptor (NLR) protein 3 (NLRP3) inflammasome activation in the onset of diabetic retinopathy (DR) using retina and vitreous from donors without diabetes mellitus (CTL), with diabetes mellitus alone (DM), and with DR. Retinal expression of glial fibrillary acidic protein (GFAP) and ionized calcium-binding adapter molecule 1 (Iba-1), the key markers of retinal inflammation, connexin43 (Cx43) which is involved in upstream inflammasome regulation, as well as NLRP3 and cleaved caspase-1, the main markers of inflammasome activation, were evaluated using immunohistochemistry and Western blotting. Vitreous interleukin (IL)-1β and IL-18, biomarkers of the activated inflammasome, were measured using a Luminex multiplex assay. Results showed a significant increase in the number and size of Iba-1^+^ cells and NLRP3 expression in DM, while a significant increase in GFAP, Cx43, cleaved caspase-1 and vitreous IL-18, as well as a further increase in Iba-1 and NLRP3 was found in DR. This suggests that the inflammasome is already primed in DM before its activation in DR. Furthermore, IL-18 may act as the major effector of inflammasome activation in DR while nuclear translocation of cleaved caspase-1 may play a role in gene transcription contributing to DR onset.

## 1. Introduction

Diabetic retinopathy (DR) is a chronic ocular complication of diabetes and the leading cause of vision loss in the working-age population [1,2]. At the beginning, microaneurysms and intra-retinal hemorrhages manifest in mild non-proliferative DR (NPDR). Increased numbers and sizes of intra-retinal hemorrhages, venous beading and vascular shunts between areas of retinal non-perfusion develop as the disease progresses to moderate and severe NPDR. This results in retinal non-perfusion and ultimately leads to neovascularization [3,4], the hall mark of proliferative DR (PDR) which can cause further sight-threatening complications such as retinal detachment and vitreous hemorrhage [4]. For patients with NPDR, current clinical management focuses on monitoring risk factors such as glycemic level, blood pressure, lipid level and smoking cessation; however, no direct treatment is available to stop disease progression [5,6,7,8]. While pan-retinal-photocoagulation, slow-release corticosteroid therapy and anti-vascular endothelial growth factor (anti-VEGF) injections are available, they are used to treat sight-threatening DR but not NPDR, and do not target the underlying disease mechanism [9]. This highlights the need for a novel therapy which specifically targets the pathogenesis of DR early on to prevent worsening of NPDR. As such, it is crucial to understand the mechanisms involved in the onset of DR.

Increasing numbers of studies have shown that the nucleotide-binding oligomerization domain-(NOD-) like receptor (NLR) protein 3 (NLRP3) inflammasome pathway is implicated in diabetes mellitus (DM) and DR [10,11,12,13,14,15,16,17,18]. The inflammasome is part of the innate immunity which triggers inflammatory cascades to eliminate harmful pathogens and dead cells from the system. It is “primed” when detection of danger signals such as damage-associated molecular patterns (DAMPs) or pathogen-associated molecular patterns (PAMPs) lead to nuclear translocation of nuclear factor kappa B (NFĸB), increasing the expression of proteins in the inflammasome pathway, including NLRP3, procaspase-1 and precursors of interleukin (IL)-1β and IL-18 in the cytosol [19,20,21]. Subsequently, the inflammasome is “assembled” when a signal, such as extracellular adenosine triphosphate (ATP), triggers the oligomerization of NLRP3, apoptosis-associated speck-like protein containing a CARD (ASC) and procaspase-1, activating the multimeric inflammasome complex [19,20,21]. This consequently leads to proteolytic cleavage of procaspase-1 to cleaved caspase-1 resulting in the cleavage and release of pro-inflammatory cytokines, IL-1β and IL-18 in their biologically active forms [19,20,21].

Preclinical studies have shown that the NLRP3 inflammasome is dysregulated in DR with significantly higher levels of aggregated NLRP3 and cleaved caspase-1 found in retinal cells in vitro and donor retinal explants ex vivo cultured under DR conditions [12,13,14]. Furthermore, inhibiting ATP-induced inflammasome activation by blocking connexin43 (Cx43) hemichannels has been shown to ameliorate the increased release of pro-inflammatory cytokines and vascular leakage in DR [12,13,14]. The Akimba mouse model, which is characterized by hyperglycemia, inflammation, and advanced DR vascular lesions also demonstrates significantly higher expression of NLRP3, ASC and caspase-1 compared to wild-type mice [16]. The use of anakinra, a monoclonal IL-1 antibody, has been shown to ameliorate IL-1β-induced accelerated retinal cell death by preventing activation of transcription factor NFĸB as well as an increase in oxidative stress and mitochondrial damage [22,23,24]. Another NLRP3 inflammasome inhibitor, MCC950, has also been shown to prevent hyperglycemia-induced retinal vascular damage, retinal thinning, retinal leakage as well as IL-1β release [25]. These studies show that NLRP3 inflammasome activation is involved in DR pathogenesis. However, DR preclinical models are generated through genetic, dietary and pharmacological modifications and do not replicate the chronicity of the disease, therefore the inflammasome data acquired from them may not fully represent the inflammasome in human DR retina [26].

Only a small number of human studies have investigated the role of the inflammasome in DR. A significant increase in vitreous IL-1β and IL-18 has previously been reported in patients with NPDR and PDR compared to controls [18,27,28,29]. However, besides Chen et al. [29], no studies have investigated their levels in patients with DM without DR, which is important to evaluate changes in the inflammasome in the transition from diabetes alone to diabetes with DR [29].

Therefore, the aim of this study was to characterize inflammasome activation leading to DR onset using retina and vitreous from non-diabetic controls (CTL), and donors with DM alone without retinopathy, and with DR. The study involved histological analysis using hematoxylin and eosin (H&E) staining, retinal expression of inflammatory markers glial fibrillary acidic protein (GFAP) and ionized calcium-binding adapter molecule-1 (Iba-1), retinal expression of key markers of inflammasome activation (NLRP3 and cleaved caspase-1), as well as vitreous levels of key inflammasome biomarkers (IL-1β and IL-18).

## 2. Results

### 2.1. Retinal Structure Was Severely Disrupted in DR

**Figure 1:**
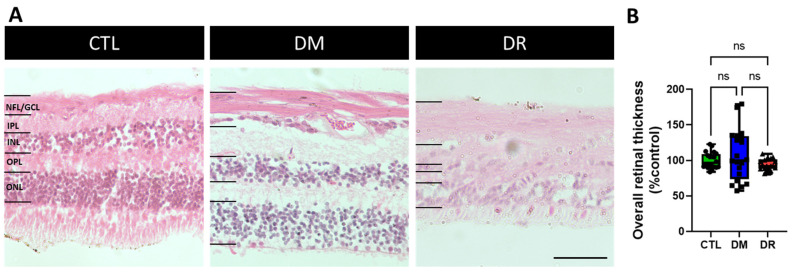
H&E staining of retinal sections demonstrating a severely disrupted retinal structure in DR compared to CTL and DM. (**A**) Images show well-defined retinal layers with high nuclear density in the INL and ONL in CTL and DM. In contrast, inter-lamellar boundaries were indistinct and nuclei in the INL and ONL were severely lost in DR. The total retinal thickness was reduced in DR compared to CTL and DM. However, the NFL appeared thicker in DR compared to CTL and DM. (**B**) The retinal thickness between CTL, DM and DR was not significantly different. Scale bar = 50 µm. NFL/GCL = nerve fiber layer/ganglion cell layer; IPL = inner plexiform layer; INL = inner nuclear layer; OPL = outer plexiform layer; ONL = outer nuclear layer; CTL = control; DM = diabetes mellitus alone; DR = diabetic retinopathy; ns = no significant difference.

All retinal layers were well-defined with high nuclear density in the inner nuclear layer (INL) and the outer nuclear layer (ONL) in CTL and DM (Figure 1A). In contrast, inter-lamellar boundaries were indistinct and nuclei in the INL and ONL were severely lost in DR, although there was no significant difference in overall retinal thickness across the groups (Figure 1B) (CTL: 100.0 ± 2.6%, DM: 107.1 ± 8.0%, DR: 93.8 ± 1.8%; CTL vs. DM: *p* = 0.9922; CTL vs. DR: *p* = 0.5043; DM vs. DR: *p* = 0.5963). However, compared to CTL and DM, the nerve fiber layer/ganglion cell layer (NFL/GCL) appeared thicker in DR (Figure 1A).

### 2.2. GFAP Expression Was Significantly Upregulated in DR

**Figure 2:**
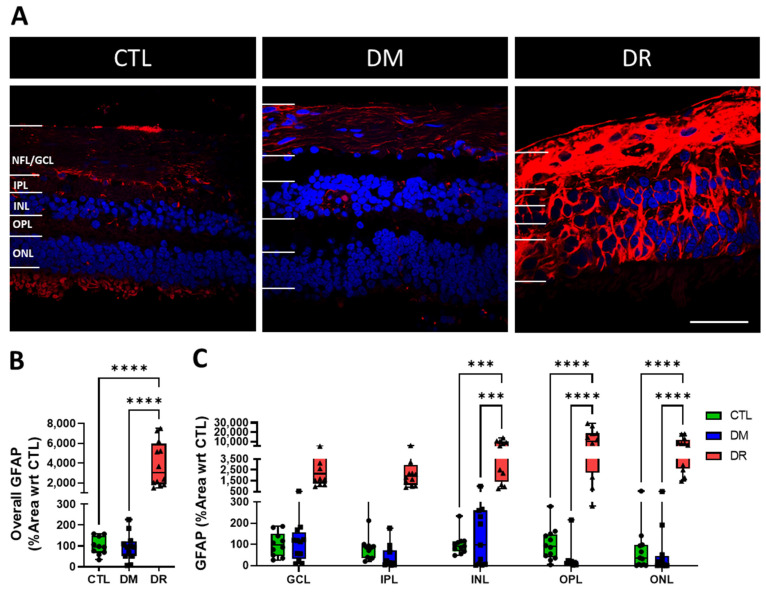
GFAP expression was significantly increased in DR. (**A**) Immunohistochemical images show that GFAP (red) was minimally expressed in CTL and DM in the NFL/GCL, IPL and INL, but significantly increased in all retinal layers in DR. (**B**) Quantification of GFAP expression in the entire retina showed low levels of GFAP expressed in CTL and DM, while significantly higher levels were found in DR compared to CTL (*p* ≤ 0.0001) and DM (*p* ≤ 0.0001). (**C**) Quantification by each retinal layer showed GFAP expression was significantly higher in DR compared to CTL and DM in the INL (*p* ≤ 0.001), OPL (*p* ≤ 0.0001) and ONL (*p* ≤ 0.0001). Scale bar = 50 μm. *** *p* ≤ 0.001; **** *p* ≤ 0.0001. Statistical analysis was carried out using one-way ANOVA for expression in the entire retina and two-way ANOVA for expression in each retinal layer. Data point of each patient in CTL, DM and DR are marked as circle, square and triangle, respectively.

In CTL and DM, GFAP was expressed at low levels and restricted within the NFL/GCL, IPL and INL. In contrast, significant GFAP upregulation was found in all layers in DR (Figure 2A). Quantification of the % area covered by GFAP labelling showed low expression levels in CTL (100.0 ± 13.6%) and DM (93.7 ± 18.7%), which increased significantly in DR (3878.1 ± 655.4%; CTL vs. DR: *p* ≤ 0.0001; DM vs. DR: *p* ≤ 0.0001) (Figure 2B). Similarly, quantification by each retinal layer demonstrated minimal GFAP expression in CTL and DM followed by significant elevation in DR in the INL (CTL: 100.0 ± 15.0%, DM: 188.3 ± 85.3%, DR: 5731.9 ± 2270.5%; CTL vs. DR: *p* ≤ 0.001; DM vs. DR: *p* ≤ 0.001), OPL (CTL: 100.0 ± 22.1%, DM: 37.8 ± 25.5%, DR: 11456.0 ± 2905.7%; CTL vs. DR: *p* ≤ 0.0001; DM vs. DR: *p* ≤ 0.0001) and ONL (CTL: 100 ± 49.1%, DM: 99.0 ± 50.4%, DR: 7720.5 ± 1685.1%; CTL vs. DR: *p* ≤ 0.0001; DM vs. DR: *p* ≤ 0.0001).

### 2.3. Iba-1^+^ Cells Increased in Number, Size, Shape and Invaded into the ONL in DR

**Figure 3:**
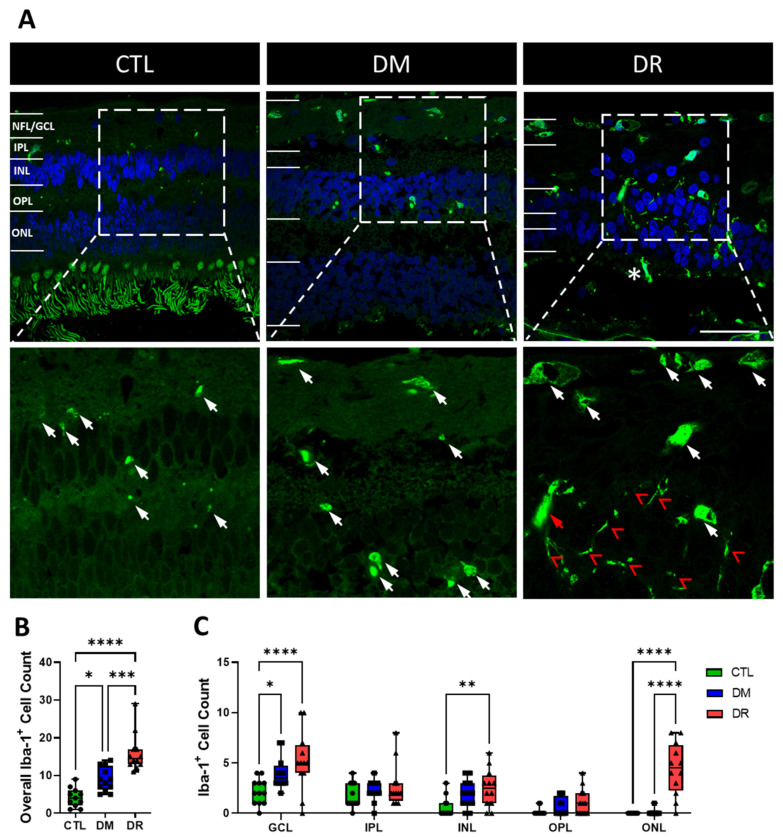
Iba1^+^ cells increased in number and size and migrated more frequently towards the ONL comparing CTL to DM to DR. (**A**) Immunohistochemical images showing the increased number and size of Iba-1^+^ cells (green, white arrows) from CTL to DM to DR. Iba-1^+^ cells in CTL and DM remained within the NFL/GCL, IPL and INL, but invaded into the ONL in DR, becoming rod-like (red arrow) and displaying long, branched dendrites (red arrowheads), while some were found in the subretinal space (asterisk) between photoreceptors and RPE. (**B**) Iba-1^+^ cell count in the entire retina was significantly higher in DM compared to CTL (*p* ≤ 0.05), and significantly higher in DR compared to CTL (*p* ≤ 0.0001) and DM (*p* ≤ 0.001). (**C**) Iba-1^+^ cell count quantified by each retinal layer showed that in the GCL, Iba-1^+^ cell count was significantly higher in DM (*p* ≤ 0.05) and DR (*p* ≤ 0.0001) compared to CTL. In the INL, Iba-1^+^ cell count was higher in DM compared to CTL but without significant difference, while the cell count was significantly higher in DR compared to CTL (*p* ≤ 0.01). In the ONL, Iba-1^+^ cell count was significantly higher in DR compared to CTL (*p* ≤ 0.0001) and DM (*p* ≤ 0.0001). Iba-1^+^ cell count in the IPL and OPL was low and showed no significant difference between CTL, DM and DR. Insets of immunohistochemical images are presented in the bottom row at 2× magnification of the top row. Scale bar = 50 µm. Statistical analysis was carried out using one-way ANOVA for expression in the entire retina and two-way ANOVA for expression in each retinal layer. * *p* ≤ 0.05; ** *p* ≤ 0.01; *** *p* ≤ 0.001; **** *p* ≤ 0.0001. Data point of each patient in CTL, DM and DR are marked as circle, square and triangle, respectively.

The number and size of Iba-1^+^ cells increased from CTL to DM to DR (Figure 3A). In CTL and DM, Iba-1^+^ cells remained within the NFL/GCL, IPL and INL, but invaded into the ONL in DR with some also found in the subretinal space (asterisk, Figure 3A) between the photoreceptor layer and the retinal pigment epithelium (RPE) layer. Compared to CTL and DM, Iba-1^+^ cells in the NFL/GCL were larger and rounder in DR while the ones invading into the ONL became rod-like (red arrow, Figure 3A) with long, branched dendrites (red arrowhead, Figure 3A).

Quantification highlighted the total number of Iba-1^+^ cells in the entire retina significantly increased from CTL (5.0 ± 1.1 cells) to DM (8.9 ± 0.9 cells; *p* ≤ 0.05), and also significantly increased from CTL and DM to DR (16.0 ± 1.5 cells; CTL vs. DR: *p* ≤ 0.0001, DM vs. DR: *p* ≤ 0.001) (Figure 3B). Quantification by each retinal layer showed that the Iba-1^+^ cell count in the NFL/GCL increased significantly from CTL (2.2 ± 0.4 cells) to DM (4.0 ± 0.5 cells; *p* ≤ 0.05) and DR (5.2 ± 0.9 cells; *p* ≤ 0.0001), and also appeared to increase from DM to DR but without a significant difference (*p* = 0.1622). Iba-1^+^ cell count in the INL appeared elevated in DM (1.9 ± 0.4 cells) compared to CTL (0.6 ± 0.5 cells) but without significant difference (*p* = 0.0941), and was significantly higher in DR (2.5 ± 0.57 cells) compared to CTL (*p* ≤ 0.01). In the ONL, Iba-1^+^ cell count was low in CTL (0.0 ± 0 cells) and DM (0.1 ± 0.9 cells) but significantly higher in DR (4.4 ± 0.8 cells; CTL vs. DR: *p* ≤ 0.0001; DM vs. DR: *p* ≤ 0.0001) (Figure 3C).

### 2.4. Cx43 Expression Increased in the GCL of DR

**Figure 4:**
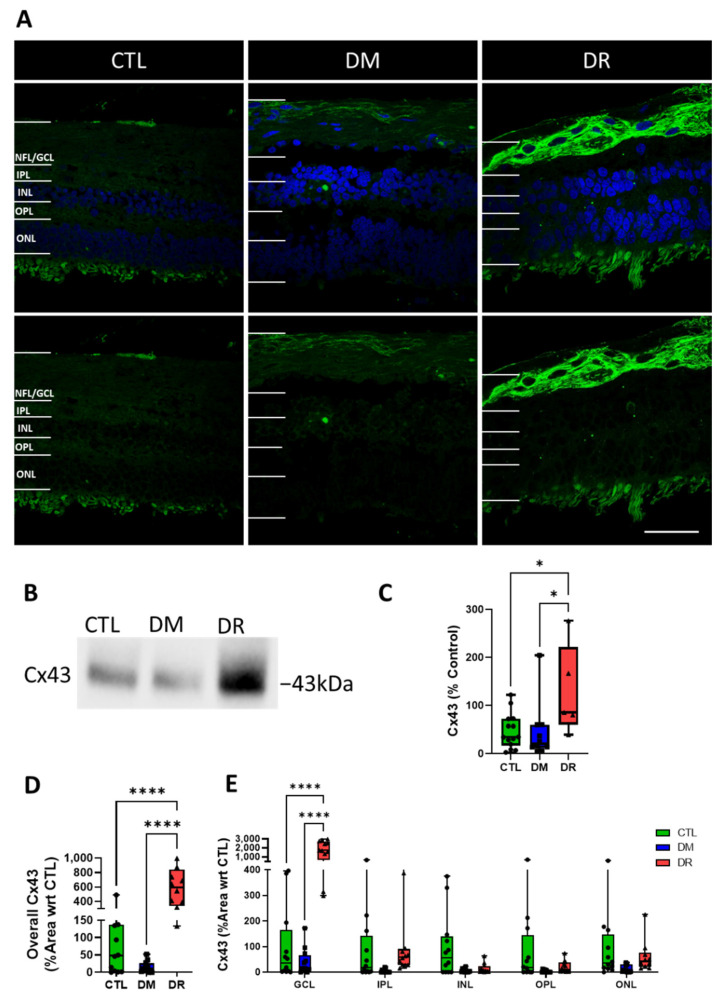
Cx43 expression increased significantly in the NFL/GCL in DR. (**A**) Immunohistochemistry labelling of Cx43 (green) showed low Cx43 expression in CTL and DM while a significant increase was found in DR, specifically in the NFL/GCL. (**B**) Western blotting analysis also showed low Cx43 levels in CTL and DM but high expression in DR. (**C**) Western blotting quantification showed lower Cx43 expression in DM compared to CTL but without significant difference (*p* = 0.9945), while significantly more Cx43 was expressed in DR compared to CTL (*p* ≤ 0.05) and DM (*p* ≤ 0.05). (**D**) A similar trend was found with Cx43 levels in the entire retina with slightly lower expression in DM compared to CTL but without significant difference (*p* = 0.8591) and significantly higher expression in DR compared to CTL (*p* ≤ 0.0001) and DM (*p* ≤ 0.0001). (**E**) Quantification of Cx43 expressed in each retinal layer highlighted significantly higher Cx43 levels in DR compared to CTL (*p* ≤ 0.0001) and DM (*p* ≤ 0.0001) but only in the GCL, while low expression levels were found in other retinal layers in all groups. Scale bar = 50 µm. Statistical analysis was carried out using one-way ANOVA for overall expression in the entire retina and two-way ANOVA for expression in each retinal layer. * *p* ≤ 0.05; **** *p* ≤ 0.0001. Data point of each patient in CTL, DM and DR are marked as circle, square and triangle, respectively.

The expression of Cx43 was minimal in CTL and DM, but significantly increased in DR, specifically in the NFL/GCL (Figure 4A,B). Western blotting analysis showed slightly lower expression in DM compared to CTL although without statistical significance (*p* = 0.9945) and significantly higher Cx43 expression in DR (129.7 ± 42.1%) compared to CTL (46.7 ± 9.7%; *p* ≤ 0.05) and DM (44.4 ± 18.9%; *p* ≤ 0.05) (Figure 4C).

Similarly, quantification of the % area covered by Cx43 in the overall retina showed lower levels of Cx43 in DM (15.6 ± 4.7%) compared to CTL (100.0 ± 44.2%) although without statistical significance (*p* = 0.8591) and significantly higher Cx43 levels in DR (584.4 ± 82.8%) compared to CTL (*p* ≤ 0.0001) and DM (*p* ≤ 0.0001) (Figure 4D). Quantification of Cx43 within each retinal layer showed significantly higher expression in DR (1734.3 ± 287.6%) compared to CTL (100.0 ± 44.2%; *p* ≤ 0.0001) and DM (40.1 ± 16.2%; *p* ≤ 0.0001) in the NFL/GCL only. On the other hand, minimal expression was found in other retinal layers with no significant difference between the groups (Figure 4E).

### 2.5. Cleaved-Caspase-1 Expression Increased in the INL and ONL in DR

**Figure 5:**
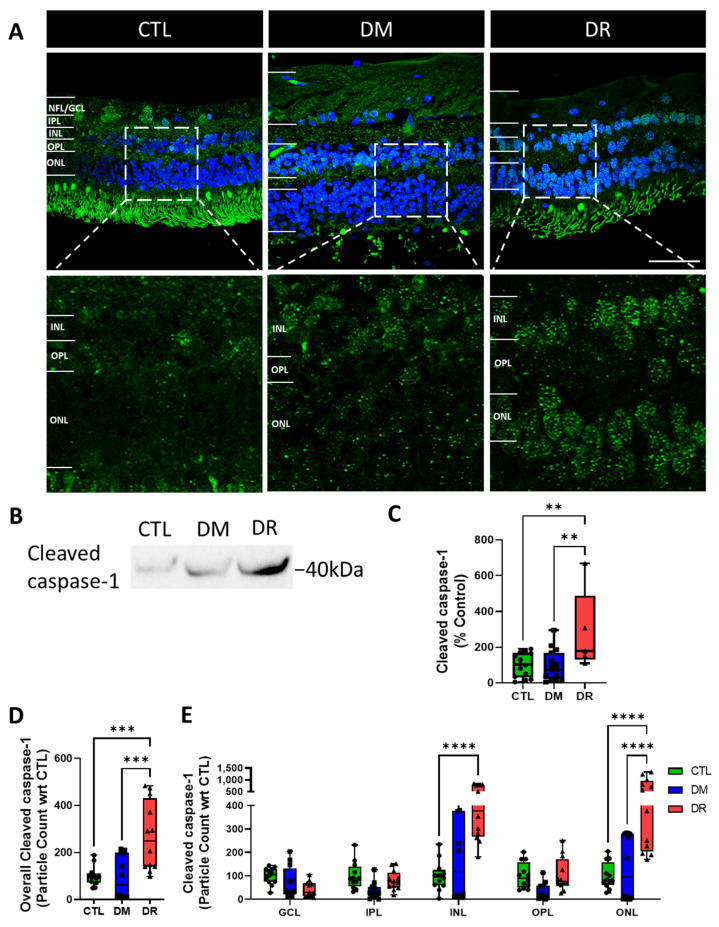
Cleaved caspase-1 significantly increased in DR in the INL and ONL. (**A**) Immunohistochemical labelling of cleaved caspase-1 showed increased expression levels from CTL to DM to DR. Specifically, expression of cleaved caspase-1 was localized in nuclei in the INL and ONL (**B**) Western blotting analysis also found increased cleaved caspase-1 levels from CTL to DM to DR. (**C**) Quantification of bands showed no difference in cleaved caspase-1 expression between CTL and DM (*p* = 0.9990) while a significant increase was found in DR compared to CTL (*p* ≤ 0.01) and DM (*p* ≤ 0.01). (**D**) Quantification of cleaved caspase-1 expressed in the entire retina appeared higher in DM compared to CTL but without significant difference (*p* = 0.9935), with significantly higher expression in DR compared to CTL (*p* ≤ 0.001) and DM (*p* ≤ 0.001). (**E**) Similarly, quantification of cleaved caspase-1 expressed in each retinal layer showed significantly higher expression in DR compared to CTL (*p* ≤ 0.0001) and DM (*p* ≤ 0.0001) in the INL and ONL, while minimal expression was found in other retinal layers across all groups. Inserts of immunohistochemical images are presented in the bottom row at 3×magnification of the top row. Scale bar = 50 µm. Statistical analysis was carried out using one-way ANOVA for expression in the entire retina and Western blotting, and two-way ANOVA for expression in each retinal layer. ** *p* ≤ 0.01; *** *p* ≤ 0.001; **** *p* ≤ 0.0001. Data point of each patient in CTL, DM and DR are marked as circle, square and triangle, respectively.

The expression of cleaved caspase-1 increased from CTL to DM to DR. Specifically, cleaved caspase-1 was localized in cell nuclei in the INL and ONL (Figure 5A). Western blotting analysis also showed increased cleaved caspase-1 expression from CTL to DM to DR. Bands were located at 40 kDa suggesting dimerization of the p20 subunits of cleaved caspase-1 (Figure 5B). Quantification of bands showed no difference in the expression in CTL (100.0 ± 17.64%) and DM (98.3 ± 21.86; *p* = 0.9990) while expression in DR (283.7 ± 101.8%) increased significantly compared to CTL (*p* ≤ 0.01) and DM (*p* ≤ 0.01) (Figure 5C). Similarly, quantification of cleaved caspase-1 in the entire retina showed significantly higher levels in DR (268.4 ± 42.3 particles) compared to CTL (100.0 ± 12.1 particles; *p* ≤ 0.001) and DM (95.4 particles ± 26.2; *p* ≤ 0.001) (Figure 5D), particularly in the nuclear layers, INL and ONL. In the INL, cleaved caspase-1 expression was significantly higher in DR (478.0 ± 72.5 particles) compared to CTL (100 ± 18.08 particles; *p* ≤ 0.0001) and DM (183.6 ± 55.6 particles; *p* ≤ 0.0001). In the ONL, cleaved caspase-1 expression was also significantly higher in DR (588.4 ± 122.8 particles) compared to CTL (1000 ± 16.1 particles; *p* ≤ 0.0001) and DM (132.8 ± 41.1 particles; *p* ≤ 0.0001) (Figure 5E).

### 2.6. NLRP3 Expression Increased in the INL and ONL in DR

**Figure 6:**
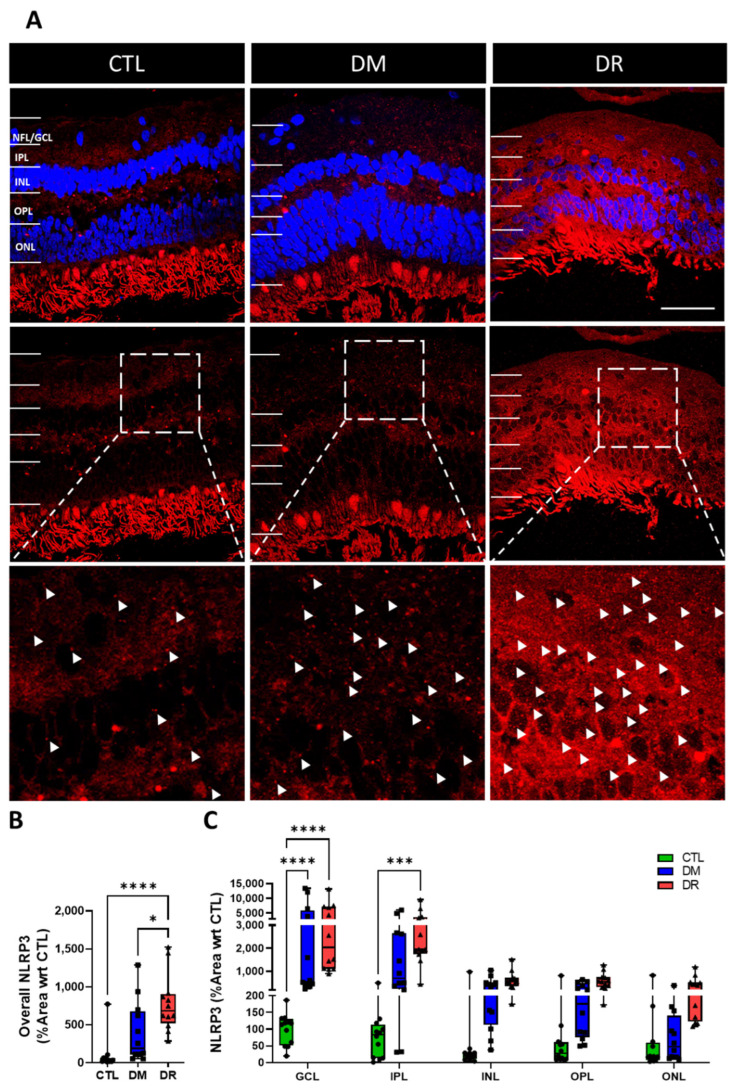
NLRP3 is upregulated in DR compared to CTL and DM. (**A**) Immunohistochemical labelling of NLRP3 (red) showed low levels of NLRP3 in CTL and DM but high levels in DR. (**B**) Quantification of levels in the entire retina showed NLRP3 expression was significantly higher in DR compared to CTL (*p* ≤ 0.0001) and DM (*p* ≤ 0.05). (**C**) Quantification by each retinal layer showed NLRP3 expression in the GCL was significantly higher in DM (*p* ≤ 0.0001) and DR (*p* ≤ 0.0001) compared to CTL. Moreover, NLRP3 expression in the IPL was significantly higher in DR compared to CTL (*p* ≤ 0.001). Minimal levels of NLRP3 were expressed in all groups in the INL, OPL and ONL. Inserts of immunohistochemical images are presented in the bottom row at 3× magnification of the middle row. Scale bar = 50 µm. Statistical analysis was carried out using one-way ANOVA for overall expression in the entire retina and two-way ANOVA for expression in each retinal layer. * *p* ≤ 0.05; *** *p* ≤ 0.001; ****p ≤ 0.0001. Data point of each patient in CTL, DM and DR are marked as circle, square and triangle, respectively.

NLRP3 expression was low in CTL and DM but became elevated in DR (Figure 6A). Quantification of the % area showed NLRP3 expression increased in the entire retina from CTL to DM to DR, with significantly higher expression found in DR (780.6 ± 109.1%) compared to CTL (100.0 ± 61.7%; *p* ≤ 0.0001) and DM (397.6 ± 116.7%, *p* ≤ 0.05) (Figure 6B). Quantification by each retinal layer showed significantly higher NLRP3 expression in the NFL/GCL in DM (3371.4 ± 1376.1%; *p* ≤ 0.0001) and DR (4072.4 ± 1117.1%; *p* ≤ 0.0001) compared to CTL (100.0 ± 13.6%). Moreover, NLRP3 expression in the IPL was significantly higher in DR (3086.3 ± 736.6%) compared to CTL (100.0 ± 38.2%; *p* ≤ 0.001). Minimal NLRP3 expression was found in the INL, OPL and ONL in all groups (Figure 6C).

### 2.7. Cx43 Expression Correlated Positively with Cleaved Caspase-1 and NLRP3 Levels

**Figure 7:**
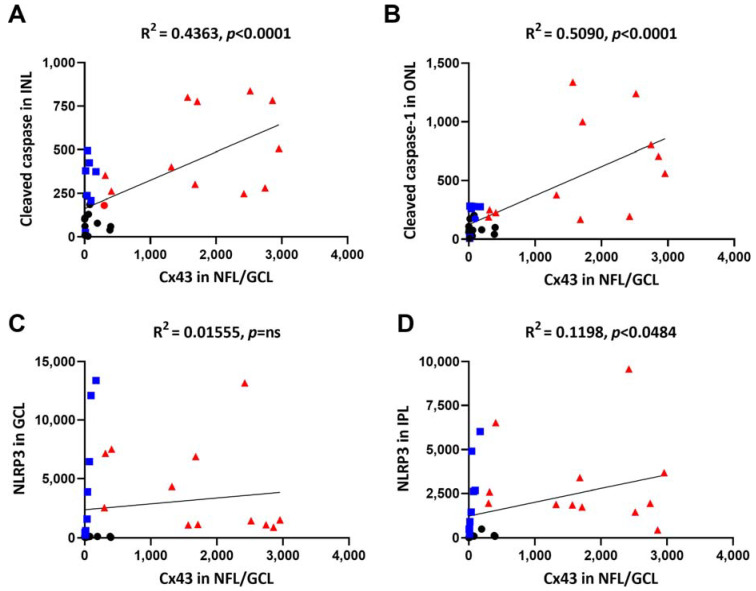
Simple linear regression between Cx43 in the NFL/GCL and cleaved caspase-1 in the INL and ONL, as well as NLRP3 in the NFL/GCL and IPL. (**A**,**B**) Cx43 expression in the NFL/GCL showed a positive linear correlation with cleaved caspase-1 expression in the INL and ONL. (**C**,**D**) Cx43 in the NFL/GCL did not correlate with NLRP3 in the NFL/GCL and showed a weak positive linear correlation with NLRP3 in the IPL; black circle = CTL, blue square = DM, red triangle = DR ns = no significant difference.

Cx43 expression in the NFL/GCL showed a positive linear correlation with cleaved caspase-1 expression in the INL (Figure 7A. R^2^ = 0.4363, *p* < 0.0001) and ONL (Figure 7B. R^2^ = 0.5090, *p* < 0.0001). Cx43 in the NFL/GCL as well as cleaved caspase-1 in the INL and ONL were minimally expressed in CTL (black circles) and DM (blue squares) while increased expressions were found in DR (red triangles) (Figure 7A,B).

On the other hand, Cx43 in the NFL/GCL did not correlate with NLRP3 in the NFL/GCL (Figure 7C. R^2^ = 0.05111, *p* = 0.4892) but showed a weak positive linear correlation with NLRP3 in the IPL (Figure 7D. R^2^ = 0.1198, *p* < 0.0484). The expression of NLRP3 in the NFL/GCL and IPL remained low in CTL and showed a wide range in DM and DR (Figure 7C,D).

### 2.8. Vitreous IL-18 Levels Were Significantly Higher in DR

**Figure 8:**
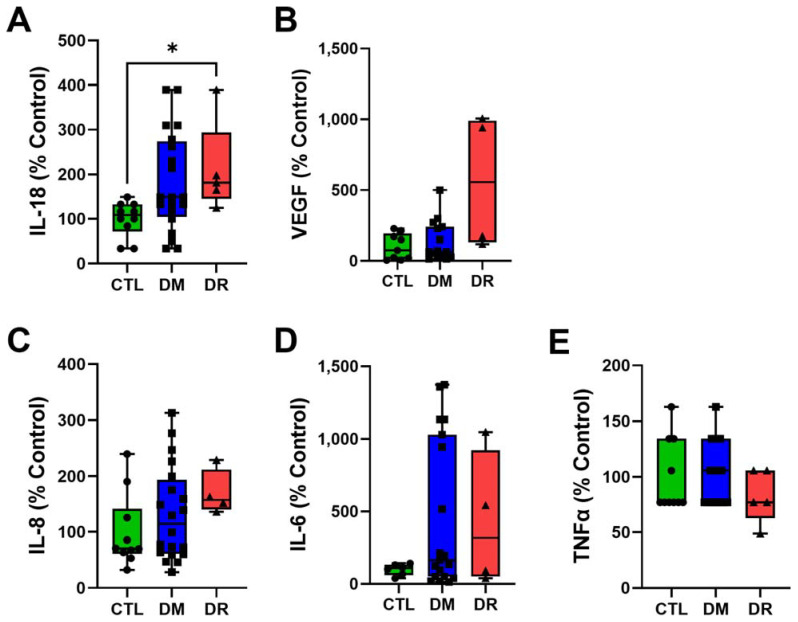
Vitreous IL-18 was the only cytokine showing a significant increase in DR compared to CTL. (**A**) Vitreous IL-18 showed a tendency to increase from CTL to DM to DR but a significant increase was only found in DR compared to CTL. (**B**,**C**) While not significantly different, vitreous VEGF and IL-8 levels appeared to increase from CTL to DM to DR. (**D**) Vitreous IL-6 levels were lower in CTL compared to DM and DR but without significant difference. (**E**) The level of TNF-α appeared lower in DR compared to CTL and DM but without significant difference. IL-1β and IL-10 were below the detectable threshold. Statistical analysis was carried out using Kruskal–Wallis test followed by post hoc Dunn’s multiple comparisons test. * *p* ≤ 0.05. Data point of each patient in CTL, DM and DR are marked as circle, square and triangle, respectively.

The level of vitreous IL-18 increased from CTL (100.0 ± 12.5%) to DM (181.6 ± 24.8%) to DR (211.8 ± 46.1%) with a significance difference found only between CTL and DR (*p* ≤ 0.05) (Figure 8A). The level of vitreous VEGF increased from CTL (100.0 ± 30.5%) to DM (137.0 ± 36.8%) to DR (560.2 ± 239.5%) but no significant difference was found between the groups (CTL vs. DM: *p* > 0.9999; CTL vs. DR: *p* = 0.1023; DM vs. DR: *p* = 0.2311) (Figure 8B). The level of vitreous IL-8 appeared to increase from CTL (100.0 ± 20.9%) to DM (132.3 ± 18.8%) to DR (170.0 ± 20.31%) but without significance between the groups (CTL vs. DM: *p* = 0.9829; CTL vs. DR: *p* = 0.2282; DM vs. DR: *p* = 0.6630) (Figure 8C). The level of vitreous IL-6 was much lower in CTL (100.0 ± 16.0%) compared to DM (454.5 ± 118.3%) and DR (430.6 ± 234.9%) but no significant difference was found between the groups (CTL vs. DM: *p* = 0.9240; CTL vs. DR: *p* > 0.9999) (Figure 8D). The level of vitreous TNF-α was similar in CTL (100.0 ± 10.3%) and DM (101.2 ± 6.3%) and slightly lower in DR (82.9 ± 10.7%) but no significant difference was found between groups (CTL vs. DM: *p* > 0.9999, CTL vs. DR: *p* > 0.9999; DM vs. DR: *p* = 0.7233) (Figure 8E). Vitreous IL-1β and IL-10 levels were below the detectable threshold.

## 3. Discussion

Early cessation of DR progression is crucial to protect patients from vision loss in PDR but currently there is no treatment that can fully stop DR progression by targeting the underlying DR pathogenesis. Involvement of the NLRP3 inflammasome in DR has been established previously and in order to understand the mechanism in early DR pathogenesis, the aim of this study was to characterize activation of the NLRP3 inflammasome leading to DR onset using donor retinas and vitreous from CTL, DM and DR.

An increase in GFAP occurs when intermediate filaments are upregulated in Müller cells in response to stress and this has previously been reported in inherited retinal degeneration, chronic retinal diseases, as well as retinal trauma induced by ischemia or laser damage [30]. Results in this study revealed minimal GFAP expression within the NFL, IPL and INL in CTL and DM, but significant upregulation in all retinal layers in DR. The same pattern has been shown in previous in vivo DR models in which GFAP was only elevated in the retina of diabetic mice exposed to pro-inflammatory cytokines but not in diabetic mice alone [31,32,33]. It was interesting to note that Müller cell gliosis only occurred in DR but not in DM, which indicates that only the retina in DR was under inflammatory stress despite hyperglycemia being present in both conditions.

Next, we investigated the expression of Iba-1^+^ cells, which represent activated microglia and macrophages. Results showed Iba-1^+^ cells significantly increased in number and size from CTL to DM. In DR, the Iba-1^+^ cell count increased further and cells changed their shape, which may represent transformation from the anti-inflammatory (M2) to the pro-inflammatory (M1) phenotype [34]. Compared to GFAP, which was only elevated in DR, changes in Iba-1^+^ cells were already detected in DM, suggesting that activation of microglia and macrophages occurs prior to the activation of Müller cells. While Iba-1^+^ cells in CTL and DM were restricted to the NFL/GCL, IPL and INL, cells in DR invaded into the ONL and some were also seen in the subretinal space between the photoreceptors and the RPE that forms the outer blood-retinal-barrier. Iba-1^+^ cells in the subretinal space were likely activated resident retinal microglial/macrophages that invaded through the ONL, but these cells may have also travelled from the choroid into the subretinal space, breaching the outer blood-retinal-barrier. This is supported by previous studies showing increased permeability and disruption in inter-cellular tight junctions in RPE cells exposed to DR conditions in vitro [35], as well as pores allowing transcellular migration of Iba-1^+^ cells through the RPE in diabetic mice in vivo [36]. More studies are required to determine the origin of subretinal Iba-1^+^ cells in DR. Nevertheless, it is clear that activation of Iba-1^+^ cells is involved in the DR pathogenesis and occurs already in DM.

We also evaluated the expression of Cx43, which has been shown to be involved in upstream regulation of the inflammasome pathway in DR [14,31] with its upregulation demonstrated in cells, mouse models of DR and DR donor tissues [18]. In this study, we found minimal Cx43 expressed in CTL and DM which increased significantly in DR, specifically in the NFL/GCL.

Importantly, expression of NLRP3, a key component of the NLRP3 inflammasome complex, increased from CTL to DM to DR in the entire retina, showing significant elevation in DR compared to CTL and DM. In the GCL, the expression of NLRP3 was significantly higher in DM and DR compared to CTL, suggesting the inflammasome is “primed” in the GCL in DM before the onset of DR.

**Figure 9:**
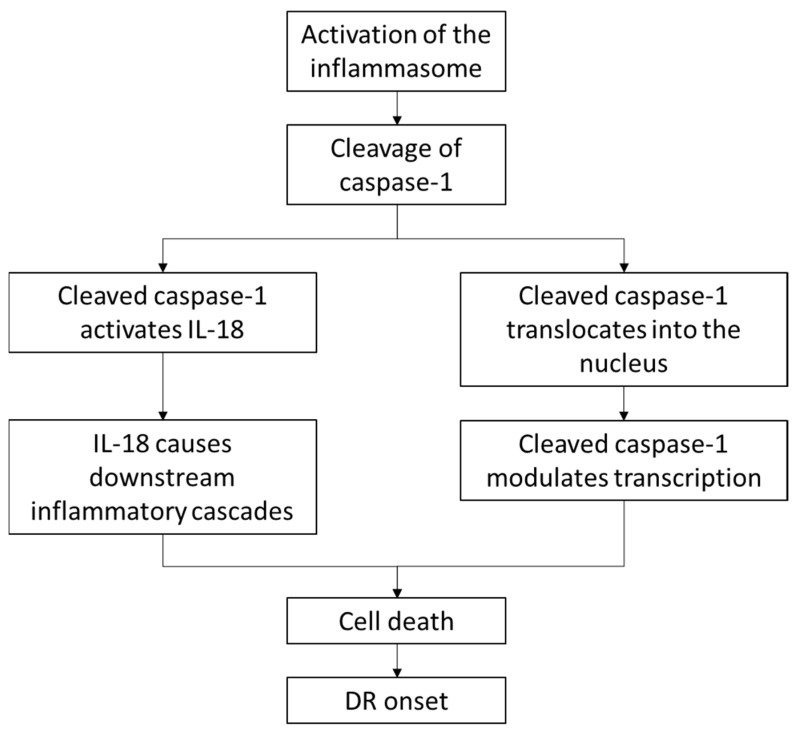
Following activation of the NLRP3 inflammasome, caspase-1 is cleaved. Cleaved caspase-1 can promote DR onset by activating IL-18 or can be translocated into nuclei in the INL and ONL, and play a role in gene transcription contributing to DR onset.

Similarly, results also showed significantly higher levels of cleaved caspase-1, a key marker of the activated inflammasome, in DR compared to CTL and DM. However, no significant difference was found between CTL and DM. This suggests “assembly” of the inflammasome occurs in DR, which is in line with inflammation in DR shown by the expression of GFAP and Iba-1. In previous mouse DR models, expression of cleaved caspase-1 was found in the plexiform layers [16], however, our results showed cleaved caspase-1 expression was specifically localized in nuclei in the INL and ONL. To our knowledge, no study has reported nuclear translocation of cleaved caspase-1 in DR. Previous studies have shown nuclear translocation of cleaved caspase-1 in apoptotic cells in vitro, but these studies were not performed under DR conditions [37,38]. Others have also suggested a role of cleaved caspase-1 in modulating gene transcription factors [39,40], including cleavage of peroxisome proliferator-activated receptor γ (PPARγ) [40] which regulates the receptor gene of VEGF, the potent pro-inflammatory cytokine that promotes neovascularization in PDR [41]. Therefore, nuclear translocation of cleaved caspase-1 may contribute to DR onset via inducing cell death and modulating various transcription factors (Figure 9). However, more studies are required to evaluate the role of nuclear translocation of cleaved caspase-1 in promoting cell death and inflammation at the onset of DR.

Furthermore, expression of Cx43 in the NFL/GCL was positively correlated with cleaved caspase-1 in the INL and ONL, all showing minimal expression in CTL and DM which increased in DR, indicating the inflammasome is activated in DR. In contrast, no distinct trend was found between expression of Cx43 in the NFL/GCL and expression of NLRP3 in the NFL/GCL or IPL. This was due to the wide range of NLRP3 expression level in DM and DR, again suggesting that the inflammasome may be “primed” in DM then activated in DR.

Additionally, we examined the levels of vitreous IL-1β and IL-18, key inflammasome biomarkers, in donor vitreous. Our study showed a tendency for vitreous IL-18 to increase from CTL to DM to DR, but a significant increase was only found in DR, but not in DM, compared to CTL. Interestingly, vitreous IL-1β was below the detection threshold in all groups. The large difference in vitreous IL-1β and IL-18 levels was unexpected as they are both activated following cleavage by active caspase-1. Different to our study, Chen et al. [29] demonstrated a significant increase in both vitreous IL-1β and IL-18 levels in NPDR compared to CTL, with no significant difference found between NPDR and DM [29]. On the other hand, Loukovaara et al. [18] showed a significant elevation in vitreous IL-18 but not IL-1β in NPDR compared to PDR; however, the study did not evaluate changes in CTL and DM [18]. Arend, Palmer and Gabay [42] explained that the release of IL-1β and IL-18 is independent from each other because pro-IL-18 is already abundantly expressed in cells and therefore regulation of active IL-18 relies only on the cleavage by active caspase-1. On the other hand, the release of active IL-1β requires the production of pro-IL-1β following active caspase-1 cleavage. Further validating our results, Zhu and Kanneganti [43] showed that IL-18 levels were increased and sustained after inflammation while IL-1β levels were increased but not sustained [32]. The currently known inflammasome/caspase-1 pathway in DR involves activation of IL-1β and IL-18 into their active forms, causing downstream inflammation and subsequently leading to cell death. However, our results suggest that instead of IL-1β, IL-18 could be the main effector of activated inflammasomes in DR. This could potentially explain the lack of efficacy of canakinumab, a human monoclonal antibody targeting IL-1β, in resolving neovascularization in patients with PDR [44]. Doyle et al. [45] demonstrated anti-inflammatory and anti-angiogenic properties of IL-18 in the retina in a mouse model of age-related macular edema; however, the effect of IL-18 in DR has not been investigated. Further investigation of the role of IL-18 leading to DR onset is therefore required.

In summary, a significant increase in the number and size of Iba-1^+^ cells and NLRP3 expression in the GCL was found in DM, while a significant increase in the expression of GFAP, Cx43 and vitreous IL-18, as well as a further increase in Iba-1 and NLRP3 was found in DR. This suggests that the inflammasome is primed in DM and activated leading to inflammation in DR. Results suggest that IL-18 may be the major effector of inflammasome activation in DR with cleaved caspase-1 expression localized in nuclei in the INL and ONL in DR donor retina, which has not been found in DR animal retina previously. Nuclear translocation of cleaved-caspase-1 may play a role in modulating transcription factors which promote cell death, leading to DR onset.

Using donor tissues increased the clinical translatability of this study compared previous studies investigating the inflammasome in DR animal models. Comparisons between CTL, DM and DR groups allowed identification of early changes leading to DR onset. It should be noted though that this is a cross-sectional study which does not account for inter-donor variations. While post-mortem time may affect the study results, there was no significant differences between CTL, DM and DR donor tissue collection time (Supplementary Figure S1). Nevertheless, this study shows that the inflammasome is implicated in early changes in DR and therefore serves as a potential target for novel therapies dedicated to prevent early DR from progressing to advanced DR.

## 4. Materials and Methods

### 4.1. Human Donor Eye Tissues and Ethics Approval

Donor retina and vitreous were obtained from the New Zealand National Eye Bank with ethics approval (NTX/06/19/CPD/AM07). Age and sex-matched donor tissues from both eyes of 8 controls without systemic or ocular diseases (CTL), 10 donors with type 2 diabetes mellitus without retinopathy (DM), as well as 4 donors with retinopathy (DR) were used. There was no significant difference in the mean age of donors with CTL (67.2 ± 8.1 years), DM (65.3 ± 14.8 years) and DR (65.3 ± 13.2 years). There was also no significant difference in the mean post-mortem time between groups (CTL 21.3 ± 5.8 h, DM 22.8 ± 3.8 h, DR 17.4 ± 8.0 h). Detailed donor history is outlined in Table S1.

### 4.2. Donor Tissue Processing for H&E Staining and Immunohistochemistry

Donor eyecups with intact sclera were dissected through the optic nerve head using a surgical blade. One half was fixed in 10% formalin for 24 h, then transferred into 70% ethanol before being paraffin-embedded, then sectioned using a microtome into 5 µm slices and mounted onto glass slides.

### 4.3. Donor Tissue Processing for Western Blotting

The other half of donor eyecups was separated into retina, choroid and vitreous and stored at −80 °C until further use. Donor eyes without sclera were also separated into retina, choroid and vitreous and stored at −80 °C until further use.

### 4.4. Deparaffinization

Paraffin sections were immersed in 100% xylene for 6 min twice then immersed in 100% ethanol for 5 min twice. Sections were then washed under running tap water for 10 min. Sections were then used for either H&E or immunohistochemistry as outlined below.

### 4.5. H&E Staining

Deparaffinized slides were stained in GILL 2 hematoxylin solution for 45 s then thoroughly washed under running tap water to remove any excess dye. Next, slides were dipped twice in 1% acidic alcohol, immersed in 1% lithium carbonate for 25 s, dipped in Eosin Y solution 10 times, then washed under running tap water. Subsequently, sections were dipped in 100% ethanol 10 times twice, then dipped 10 times in 100% xylene twice. Finally, slides were coverslipped using dibutylphthalate polystyrene xylene (DPX) as the mounting medium. Slides were visualized using a brightfield light microscope (Leica microsystems. Inc, Morrisville, NY, USA).

### 4.6. Retinal Thickness Quantification

Total retinal thickness was measured using ImageJ 1.53 software (National Institutes of Health, Bethesda, MD, USA). Three H&E images were selected from each group and three measurements were taken for each image. Each retinal thickness measurement was normalized to the mean thickness of the control group. 

### 4.7. Immunohistochemistry

For antigen retrieval, deparaffinized sections were immersed in citrate buffer (10 mM tri-sodium citrate buffer containing 0.05% Tween-20 at a pH of 6.0) and placed in a pressure cooker. Next, sections were washed in phosphate-buffered saline (PBS) 5 times for 5 min, then blocked in PBS containing 0.1% Triton and 10% normal goat or horse serum for 1 h at room temperature. The serum used for blocking was dependent on the species from which the secondary antibody was derived, therefore, goat serum was used for goat-derived antibodies while horse serum was used for donkey-derived antibodies. Subsequently, slides were dried and incubated with the primary antibodies overnight at 4 °C (Table 1). The next day, sections were washed with PBS 5 times for 5 min, then incubated with secondary antibodies (Table 1) for 2 h at room temperature in the dark. Cell nuclei were stained blue using 4′,6-diamidino-2-phenylindole (DAPI) (1:200; D9542; Sigma-Aldrich, St Louis, MO, USA) Following incubation, sections were washed in the dark with PBS 5 times for 5 min. Lastly, slides were mounted using anti-fade reagent (Citifluor™, Hatfield, PA, USA) and coverslips were sealed using nail polish.

Inflammation was detected using primary antibodies against GFAP, a common marker of Müller cell activation in diseased retina, Cx43 which is involved in upstream regulation of inflammasome activation, and Iba-1 which is specific to the cytoskeleton of activated microglia and macrophages. Activated NLRP3 inflammasome was detected using antibody against cleaved caspase-1 and NLRP3.

### 4.8. Confocal Image Acquisition and Analysis

Immunohistochemistry images were acquired using a FV1000 confocal laser scanning microscope (Olympus, Tokyo, Japan) and processed using FV-10 ASW 4.2 Viewer and quantified using ImageJ 1.53 software (National Institutes of Health, Bethesda, MD, USA). For each marker, laser power, gain and offset parameters were kept constant for all sections to allow for unbiased comparison between sections. Overall, six images were taken for each eye per donor per biomarker. The researcher was masked during image acquisition to minimize bias. During quantification, all images were converted to binary images and equal low and high threshold values were applied to all images. Depending on the type of marker, images were quantified based on either area fraction (GFAP, Cx43, NLRP3), particle count (Cleaved caspase-1), or cell count (Iba-1). Area fraction (% area) was normalized to the control group by dividing the expression in that area by the mean expression of the control group in the same area. Particle counts were carried out by measuring the number of fluorescent spots. Cell counts were carried out manually by a masked researcher. Quantification was carried out either for the entire retina from the NFL/GCL to the ONL and for each individual retinal layer. To determine whether Cx43 levels correlate with inflammasome activation, simple linear regression analyses was conducted between the expressions of Cx43, cleaved caspase-1, and NLRP3 in DM and DR retina using GraphPad Prism 9.3.1 (GraphPad software, San Diego, CA, USA).

### 4.9. Homogenization for Luminex Analysis and Western Blotting

Retina and vitreous were thawed on ice for 30 min, then homogenized twice using a bead mill homogenizer (Precellys^®^ Evolution, Bertin Technologies, Montigny-le-Bretonneux, France) at 5800 rpm, 3 × 15 s cycles, 30 s pause between cycles. Vitreous samples were homogenized without a lysis buffer while retinal samples were homogenized in a lysis buffer containing radioimmunoprecipitation assay (RIPA) buffer (10% sodium deoxycholate, 100 mM Tris at pH 8.0, 10% sodium dodecyl sulfate (SDS) and a tablet of protease and phosphatase inhibitor (#A32961, Thermo Fisher Scientific Inc., Waltham, MA, USA). After homogenization, samples were centrifuged at 10,000× *g* for 3 × 10 min at 4 °C.

### 4.10. Luminex Magnetic Assay

Vitreous levels of TNFα, IL-6, IL-8, IL-10, IL-18, IL-1β and VEGF were assessed using a Luminex assay (Human Premixed Multi-Analyte Kit, #LXSAHM, R&D Systems, Minneapolis, MN, USA) following manufacturer’s instructions [46]. Vitreous samples were incubated with antibody-coated microparticles in the dark for 2 h at room temperature on a horizontal orbital microplate shaker at 800 rpm. Each well was washed with wash buffer for 3 × 1 min on the shaker and incubated with a biotinylated-antibody cocktail in the dark for 1 h at room temperature. Wells were washed again before incubating with phycoerythrin (PE)-conjugated streptavidin for 30 min at room temperature on the shaker. After washing, microparticles in each well were resuspended in wash buffer before the plate was read (Luminex MAGPIX^®^ Analyzer, Luminex, Austin, TX, USA).

### 4.11. Protein Quantification

After homogenization, retinal lysate protein concentration was determined using a DC Protein Assay (#5000112 Bio-Rad Laboratories, Inc., Hercules, CA, USA) and absorbance was measured using Spectramax i3x Multi-Mode Microplate Reader (Molecular Devices, LLC., San Jose, CA, USA).

### 4.12. Western Blotting

Equivalent amounts of retinal protein (40 µg per lane) were separated by SDS-polyacrylamide gel electrophoresis using a 8–16% Mini-PROTEAN TGX stain-free gel (#4568106, Bio-Rad Laboratories, Inc., Hercules, CA, USA), then transferred onto a polyvinylidene difluoride (PVDF) membrane using a Trans-Blot Turbo Transfer System (#17001917, Bio-Rad Laboratories, Inc., Hercules, CA, USA). Non-specific binding was blocked by incubating the membrane in 5% non-fat milk dissolved in Tris Buffered Saline with Tween20 (TBST) at room temperature for 1 h, then incubated in primary antibodies at 4 °C overnight. On the next day, membranes were washed in TBST 3 times for 10 min and incubated in horseradish peroxidase-conjugated secondary antibodies for 2 h at room temperature and washed again in TBST. Details of antibodies are listed in Table 1. Enhanced chemiluminescent substrate was added to the membrane and incubated in the dark for 5 min (Pierce™ ECL Plus Western Blotting Substrate #32132, Thermo Fisher Scientific Inc., Waltham, MA, USA). Images were acquired using ChemiDoc MP Imaging System (#17001402 Bio-Rad Laboratories, Inc., Hercules, CA, USA). Image Lab 6.1 (Bio-Rad Laboratories, Inc., Hercules, CA, USA) was used to quantify the expression of the chemiluminescent blots. The total protein measurement on the stain-free gel was used as the loading control.

### 4.13. Statistical Analysis

Experimental data were analyzed using GraphPad Prism 9.3.1 (GraphPad software, San Diego, CA, USA). Protein quantifications were presented as box plots with the minimum value, 25th quartile, median, 75th quartile and the maximum value. For all tests, *p* < 0.05 was considered statistically significant. For proteins expressed in the entire retina in IHC and WB, as well as retinal thickness measured using H&E images, statistical analysis was conducted using one-way ANOVA. For IHC protein expressions in each retinal layer, statistical analysis was conducted using two-way ANOVA. These were followed by post hoc Tukey’s multiple comparisons test. For Luminex measurements of vitreous inflammatory cytokines, statistical analysis was conducted using Kruskal–Wallis test followed by post hoc Dunn’s multiple comparisons test.

## Figures and Tables

**Table 1:** Information on antibodies used in this study.

Antibodies	Origin	Antibody Type	IHC Working Dilution	WB Working Dilution	Catalogue Number	Company
GFAP-Cy3	Mouse monoclonal	Primary	1:1000	-	C9205	Sigma-Aldrich, St Louis, MO, USA
Connexin43	Rabbit polyclonal	Primary	1:2000	1:8000	C6219	Sigma-Aldrich, St Louis, MO, USA
Iba-1	Rabbit monoclonal	Primary	1:2000	-	ab178846	Abcam, Cambridge, UK
Cleaved Caspase-1	Rabbit polyclonal	Primary	1:50	1:1000	PA5-38099	Invitrogen, Thermo Fisher Scientific Inc., Carlsbad, CA, USA
NLRP3	Goat polyclonal	Primary	1:500	1:1000	ab4207	Abcam, Cambridge, UK
Goat anti Rabbit Alex 488	Rabbit polyclonal	Secondary	1:500	-	A-11034	Invitrogen, Thermo Fisher Scientific Inc., Carlsbad, CA, USA
Donkey anti Rabbit Alex 488	Donkey polyclonal	Secondary	1:500	-	A21206	Invitrogen, Thermo Fisher Scientific Inc., Carlsbad, CA, USA
Donkey anti Goat Cy3	Donkey polyclonal	Secondary	1:500	-	705165147	Jackson ImmunoResearch Inc., West Grove, PA, USA
Goat anti Rabbit HRP	Goat polyclonal	Secondary	-	1:2000	P044801-2	Agilent Dako, Santa Clara, CA, USA

## Data Availability

The data presented in this study are available within the article and in Supplementary Table S1.

## Supplementary Materials

The following supporting information can be downloaded at: https://www.mdpi.com/article/10.3390/ijms232214471/s1.
