# Supplementary material for: Characterization of NLRP3 Inflammasome Activation in the Onset of Diabetic Retinopathy
Source: Int J Mol Sci. 2022 Nov 21;23(22):14471. doi: 10.3390/ijms232214471 (PMC9697159; doi:10.3390/ijms232214471)
Supplement: Supplementary file 1 [file ijms-23-14471-s001.zip › ijms-1969609-supplementary.pdf]

Table S1: Full donor history

	Donor ID	Sclera	Sex	Age	Ethnicity	Post mortem delay (h)	Cause of Death	Systemic disease	Eye disease/ surgery	Experiment used
DR (n=4)	19-143	N	F	64	Polynesian	24+	Brain hemorrhage	DM	DR	WB, Luminex
									DR	WB, Luminex
	21-004	N	M	84	Indian	13.5	Pneumonia, brain hemorrhage	DM	DR, Pseudophakic	WB, Luminex
									DR, Pseudophakic	WB, Luminex
	21-029	N	M	59	Indian	24+	Cardiac arrest	DM	Non-proliferative DR, primary open angle glaucoma, laser treatment	WB, Luminex
									-	WB, Luminex
	19-081	Y	F	54	Māori	8	Pneumonia	-	Diabetic retinopathy, bilateral cataract surgery, vitrectomy	WB, IHC, H&E, Luminex
										WB, IHC, H&E, Luminex
DM (n=10)	19-100	Y	F	75	Caucasian	24	Pneumonia	DM	Bilateral pseudophakia	WB, IHC, H&E, Luminex
										WB, IHC, H&E, Luminex
	19-144	N	M	69	Indian	12	Stroke	DM	-	WB, Luminex
									Pseudophakic	WB, Luminex
	19-181	N	F	68	Japanese	24+	Subarachnoid hemorrhage	DM	-	WB, Luminex
									-	WB, Luminex
	19-182	N	F	57	Caucasian	24+	Intracranial hemorrhage	DM	-	WB, Luminex
									-	WB, Luminex
	20-070	N	F	82	Caucasian	24+	Sepsis	DM	-	WB, Luminex
									-	WB, Luminex
	20-071	N	M	71	Indian	24+	Pneumonia	DM	-	WB, Luminex
									-	WB, Luminex
	20-078	N	M	58	Caucasian	24+	Cardiac arrest	DM	-	WB, Luminex
									-	WB, Luminex
	20-079	N	M	82	Caucasian	24+	Pneumonia	DM	-	WB, Luminex
									-	WB, Luminex
	21-084	N	F	59	Caucasian	24+	Renal failure	DM	Pseudophakic	WB, Luminex

									Pseudophakic	WB, Luminex
	21-115	N	M	32	Caucasian	24+	Cerebrovascular accident/stroke	DM	-	WB, Luminex
									-	WB, Luminex
**CTL (n=10)**	19-117	N	F	59	Samoan	24+	Lymphoma, renal failure	-	-	WB, Luminex
									-	WB, Luminex
	21-060	N	M	68	Caucasian	24+	Metastatic prostate cancer	-	-	WB
									-	WB
	21-086	N	M	68	-	21	Hypoxic-ischemic encephalopathy	-	-	WB, Luminex
									-	WB, Luminex
	19-180	N	F	60	Caucasian	24+	Subarachnoid hemorrhage	-	-	WB, Luminex
									-	WB, Luminex
	20-176	N	F	78	Caucasian	24+	Subarachnoid hemorrhage	-	-	WB, Luminex
									-	WB, Luminex
	19-179	N	M	58	Caucasian	24+	Intracranial hemorrhage	-	-	WB, Luminex
									-	WB, Luminex
	18-149	N	F	80	Caucasian	24+	Heart failure	-	Pseudophakic	WB, Luminex
									Pseudophakic	WB, Luminex
	19-147	N	M	59	Caucasian	24+	Aspiration pneumonia	-	-	WB, Luminex
									-	WB, Luminex
	19-108	Y	M	73	Caucasian	6	Myelodysplastic syndrome	-	-	IHC, H&E
	19-110	Y	F	69	Caucasian	17.5	Pneumonia	-	-	IHC, H&E

N = no; Y = yes; M = male; F = female; CTL = control; DM = diabetes mellitus only without retinopathy; DR = diabetic retinopathy; WB = Western Blotting, IHC = immunohistochemistry, H&E = Hematoxylin and Eosin

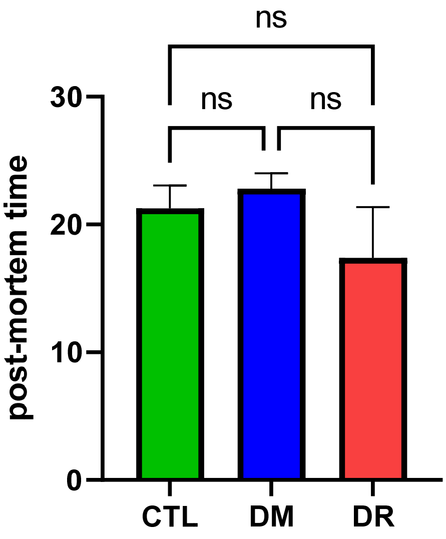

Figure S1: No significant difference was found between the post-mortem time between CTL, DM and DR; CTL = control; DM = diabetes mellitus only without retinopathy; DR = diabetic retinopathy.
